# Belowground mutualists modulate growth and aboveground defense in potato: insights from mycorrhizal and entomopathogenic nematode interactions

**DOI:** 10.1007/s00425-025-04877-w

**Published:** 2025-11-25

**Authors:** Lucía Martín-Cacheda, Xoaquín Moreira, Víctor Manuel Rodríguez, Gabriela Quiroga, Gregory Röder, Rubén Blanco-Pérez

**Affiliations:** 1 Misión Biológica de Galicia (MBG-CSIC), Apdo. 28, 36080 Pontevedra, Spain; 2 Centro de Investigaciones Agrarias de Mabegondo (CIAM), Apdo. 10, 15080 A Coruña, Spain; 3 Institute of Biology, University of Neuchâtel, Rue Emile‑Argand 11, 2000 Neuchâtel, Switzerland

**Keywords:** Arbuscular mycorrhizal fungi, Growth–defense trade-off, *Heterorhabditis bacteriophora*, Plant–soil interactions, *Rhizophagus irregularis*, *Steinernema carpocapsae*, Volatile organic compounds

## Abstract

**Main conclusion:**

Soil application of entomopathogenic nematodes enhanced the growth and modulated the aboveground defenses in potato plants, while interactions with mycorrhizal fungi shaped the volatile emissions and herbivory, underscoring the nematodes as primary drivers in multi-mutualistic systems.

**Abstract:**

Plants frequently engage with multiple belowground mutualists simultaneously, yet the outcomes of such multi-partner associations for plant traits and herbivore resistance remain poorly understood. In this study, we investigated the independent and combined effects of arbuscular mycorrhizal fungi (AMF; *Rhizophagus irregularis*) and entomopathogenic nematodes (EPNs; *Steinernema carpocapsae* and *Heterorhabditis bacteriophora*) on the growth and defense phenotype of potato (*Solanum tuberosum*) plants. Using a fully factorial experimental design, we assessed the plant height, foliar phenolic content, constitutive volatile organic compound (VOC) emissions, and leaf damage by chewing herbivores. We found that EPNs alone enhanced plant height and reduced flavonoid concentrations, consistent with a potential shift in growth–defense allocation. AMF inoculation had no significant main effects on any measured trait but interacted with EPNs in a species-specific manner. Notably, mycorrhization increased VOC emission only in the presence of *H. bacteriophora*, while co-inoculation with AMF and *S. carpocapsae* significantly reduced herbivore damage—a response not observed in either single-symbiont treatment. These findings highlight the non-additive and context-dependent nature of belowground mutualist interactions, with distinct outcomes governed by the identity of the EPN. To our knowledge, this is among the first documented cases where aboveground herbivory is attenuated through a context-specific interaction between two root-associated mutualists. Overall, our results highlight the ecological significance of considering species-specific, multi-mutualist interactions in shaping plant traits and suggest that combining functionally distinct symbionts may offer a novel approach to enhancing crop resilience in sustainable agriculture.

**Supplementary Information:**

The online version contains supplementary material available at 10.1007/s00425-025-04877-w.

## Introduction

Mutualistic interactions are foundational in structuring terrestrial ecosystems, influencing species interactions and plant traits, and driving broader community dynamics across trophic levels (Bascompte 2019; Chomicki et al. 2019). Although much of the early work in this field concentrated on isolated, pairwise relationships between plants and their symbionts, an emerging body of research reveals that plants frequently engage with multiple mutualists concurrently. These simultaneous associations give rise to intricate networks that span both, below- and aboveground systems, leading to ecological outcomes that are highly context-dependent and hardly predictable (Bronstein 2009; Guimarães et al. 2017). The presence of multiple mutualists can result in synergistic or non-additive effects on plant fitness and function, with implications that ripple across ecological communities and influence evolutionary trajectories (Yule et al. 2020; Afkhami et al. 2021; Tsiknia et al. 2021). Despite these insights, the mechanistic bases of such complex interactions—and their cascading effects on multitrophic processes—remain a critical gap in our understanding.

Arbuscular mycorrhizal fungi (AMF) and entomopathogenic nematodes (EPNs) are two ecologically important yet functionally distinct groups of belowground mutualists interacting with plants in markedly different ways. AMF are obligate biotrophs that form widespread symbiotic associations with plant roots, facilitating the uptake of essential nutrients—particularly phosphorus—and often enhancing plant growth and root development (van der Heijden et al. 1998; Martin et al. 2017; Wipf et al. 2019; Begum et al. 2019). However, the outcome of this symbiosis is context-dependent and can range from mutualistic to parasitic, especially under nutrient-rich conditions where the cost of carbon allocation to the fungus may outweigh the benefits for the plant (Johnson et al. 1997; Werner and Kiers 2015). In contrast, EPNs are insect-parasitic organisms with their symbiotic bacteria, which produce toxins to kill insect hosts (Tarasco et al. 2023). Although EPNs are not obligate mutualists of plants, they contribute to plant protection by infecting and killing herbivorous insect pests, thereby indirectly influencing plant performance and defense expression (Blanco-Pérez et al. 2024).

While AMF and EPNs do not interact directly, they may influence each other through plant-mediated processes in the shared rhizosphere. AMF colonization can modify root architecture and increase plant biomass (de Vries et al. 2021), thereby affecting root exudation and rhizosphere conditions (Bais et al. 2006; Ma et al. 2022)—factors that may alter the habitat suitability and prey dynamics for EPNs (Grewal et al. 1994). In addition, AMF are known to modulate plant defense signaling pathways, particularly those governed by jasmonic acid and salicylic acid (Fernández et al. 2014), which influence both direct defenses (e.g., secondary metabolites) and indirect defenses such as volatile organic compound (VOC) emission (Song et al. 2013; Meier and Hunter 2021). These VOCs can affect herbivore behavior and attract natural enemies (Rasmann et al. 2005; Kessler and Heil 2011), potentially increasing the effectiveness of EPNs by making herbivores more vulnerable or altering their feeding behavior. Conversely, EPN infection of insect herbivores can trigger systemic plant responses. Plants exposed to EPN-infected cadavers exhibit elevated levels of defense-related hormones, such as salicylic acid and jasmonic acid, and altered plant VOC emissions that enhance the attraction of natural enemies of herbivores (Wang et al. 2024). Moreover, EPN-infected cadavers emit characteristic VOC blends that can prime the induction of plant defense, suggesting a role in belowground-to-aboveground signaling (Helms et al. 2019). These physiological changes may, in turn, influence AMF colonization and function, as alterations in carbon allocation or cross-talk can affect the stability and mutualistic benefits of the AMF–plant symbiosis (Crosino and Genre 2022). Together, these trait-mediated feedbacks suggest that AMF and EPNs can indirectly influence each other via plant physiological and chemical pathways. Such belowground interactions may result in synergistic or antagonistic outcomes, depending on environmental conditions and the composition of soil and plant-associated communities. Understanding the context-dependent nature of these multi-mutualist interactions is crucial for predicting their ecological consequences and harnessing their potential in sustainable agriculture.

In this study, we investigated the independent and interactive effects of AMF and EPNs on plant growth, constitutive direct and indirect defense traits, and foliar herbivory in potato (*Solanum tuberosum* L.) plants. In a factorial field experiment, we manipulated the presence/absence of AMF inoculation (*Rhizophagus irregularis*) and EPN occurrence (via infected insect cadavers containing *Steinernema carpocapsae* or *Heterorhabditis bacteriophora*) to assess how these belowground mutualists influence plant growth (total height), foliar chemistry (leaf phenolic compounds as a measure of direct defense), and VOC emission patterns as indicators of indirect defense. We also examined whether changes in these plant traits translate into altered resistance to chewing herbivores. We specifically asked: (i) Do AMF and EPNs interact to influence plant traits and herbivory? (ii) Are their combined effects merely additive, or do they reflect interactive dynamics? (iii) What underlying plant traits mediate the influence of belowground mutualists on aboveground herbivory? We hypothesized that AMF and EPNs would jointly influence plant resistance, either synergistically, by enhancing the expression of plant defense traits, or compensatory, with EPNs directly reducing herbivore pressure and thereby decreasing the plant’s reliance on internal defenses, allowing for increased investment in growth. Overall, this study aims to advance our understanding of how multiple belowground mutualists modulate aboveground multitrophic interactions in crop-associated plant–herbivore systems.

## Materials and methods

### Study system

Potato (*Solanum tuberosum* L.) is one of the most widely cultivated and economically important crops worldwide (FAOSTAT 2023). It is attacked by a broad range of herbivores, including both specialists (e.g., *Leptinotarsa decemlineata*) and generalists (e.g., *Spodoptera exigua*, *Myzus persicae*), and defends itself through the production of phenolic compounds (Friedman 2006; Kumar et al. 2016) and herbivore-induced VOCs involved in indirect resistance and plant-to-plant signaling (Vázquez-González et al. 2022; Martín-Cacheda et al. 2023). Potato roots are naturally colonized by AMF species such as *R. irregularis*, which have been shown to promote growth and modulate resistance to chewing herbivores (Buysens et al. 2017; Schoenherr et al. 2019). Although AMF–plant interactions can vary depending on environmental conditions and genotype combinations, sometimes resulting in reduced plant benefit or non-cooperative outcomes, members of the Glomeraceae family, including *R. irregularis*, typically exhibit a mutualistic phenotype under agricultural conditions (Kaur et al. 2022; Yang et al. 2025). Moreover, previous studies conducted within the same experimental system have reported consistent mutualistic benefits, such as improved nutrient status and reduced disease severity (Carrara et al. 2023; Deja-Sikora et al. 2023), supporting the use of *R. irregularis* as a suitable inoculant in this study. However, these benefits do not always result in reduced herbivory (Moreira et al. 2024). Likewise, the EPN species *S. carpocapsae* and *H. bacteriophora* have been shown to enhance systemic plant resistance and trigger priming effects in neighboring plants via root-associated cues (Helms et al. 2019).

### Plant material, mycorrhization, and nematode cultures

In March 2024, we sowed 240 *S. tuberosum* tubers corresponding to three potato varieties (Kennebec, Baraka, and Desiree; 80 plants per variety) in 4-L pots filled with a 1:1 sand/peat mixture. The peat substrate (Gramoflor GmbH & Co. KG Produktion, Vechta, Germany) had a pH (CaCl₂) ranging from 4.5 to 7.5 and nutrient values of NPK 14–10–18. The sand, predominantly siliceous, had a pH between 6 and 7. We selected potato cultivars commonly grown in the study area (Pontevedra, Galicia, Spain), where they are widely commercialized as seed potatoes. Including multiple cultivars allowed us to capture a broader representation of *S. tuberosum* and to improve the generalizability of our findings beyond a single genotype. Before planting, pots were disinfected with NaClO, and the substrate was sterilized by autoclaving in double bags for three consecutive days. Following Moreira et al. (2024), mycorrhization inoculation with *R. irregularis* was performed at sowing. Half of the pots received 5 g of a commercial AMF inoculum (Atens Biotech, Tarragona, Spain), while the control group received 10 mL of a 20–30 g filtrate derived from the same inoculum, containing the associated microbial community but devoid of AMF propagules.

Two EPN populations, *S. carpocapsae* All and *H. bacteriophora* RM-102, were tested (Table 1). Following Vicente-Díez et al. (2021), both EPN species were cultured in last-instar *Galleria mellonella* (Lepidoptera: Pyralidae) reared at the Institute of Grapevine and Wine Sciences (ICVV, Logroño, Spain). Emergent infective juveniles (IJs) were collected in tap water, stored at 14 °C in darkness, and used within two weeks of emergence (Woodring and Kaya 1988). EPNs were applied to the pots as pre-infected insect larvae, prepared separately for each nematode population. To do this, groups of 10 *G. mellonella* larvae were placed in Petri dishes lined with Whatman no. 1 filter paper (in duplicate) and exposed to a 1 mL suspension containing 960 IJs. Once infection was confirmed by characteristic changes in color and texture of the dead larvae, 12 infected cadavers were introduced into the soil of each pot at sowing, concurrently with AMF inoculation, to avoid any potential impact on plant responses resulting from differing timing of inoculation. Control treatments consisted of either freeze-killed larvae or no larvae.

**Table 1:** Evaluated entomopathogenic nematode (EPN) and associated symbiotic bacterial species, with corresponding ITS GenBank accession numbers (GB Acc. No.)

EPN species	Population	GB Acc. No	Bacterial species	GB Acc. No
*Steinernema carpocapsae*	ALL	MW574913	*Xenorhabdus nematophilus*	MW574906
*Heterorhabditis bacteriophora*	RM-102	MW480132	*Photorhabdus laumondii* subsp. *laumondii*	MW574908

After sowing and applying the respective AMF and EPN treatments, potato plants were grown in a greenhouse (42.41° N, 8.64° W, Pontevedra, Spain) under controlled conditions: A minimum of 10 h of light per day (photosynthetically active radiation: 725 ± 19 μmol m⁻^2^ s⁻^1^) and temperatures maintained at 10 °C during the night and 25 °C during the day. Plants were watered three times a week.

### Experimental design and plant measures

**Fig. 1:**
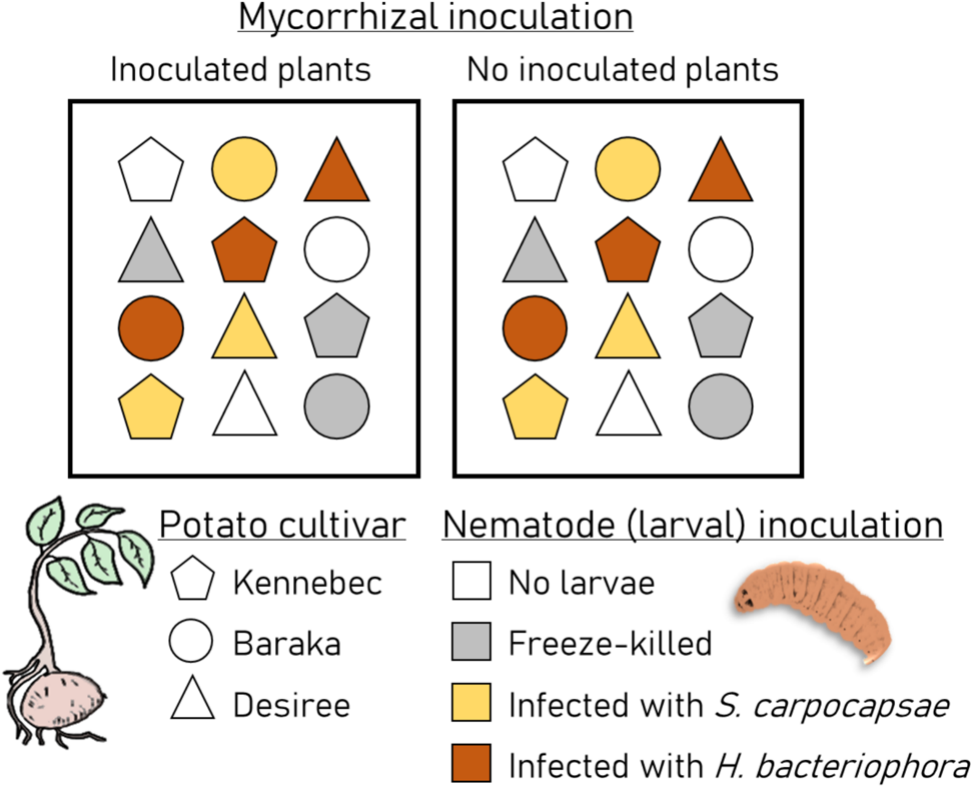
Schematic representation of a single block in the experimental design. Mycorrhizal inoculation was applied as the whole-plot factor, nematode inoculation as the split-plot factor, and genetic entry (e.g., cultivar) of potato (*Solanum tuberosum*) plants as the split-split-plot factor

A randomized split-split plot design was implemented with 10 blocks to ensure replication. The whole-plot factor was AMF inoculation (two levels: control and mycorrhization), the split-plot factor was EPN inoculation (four levels: no larvae, freeze-killed larvae, and larvae infected with *S. carpocapsae* or *H. bacteriophora*), and the split-split factor was plant variety (three levels: Kennebec, Baraka, and Desiree; Fig. 1). Blocking was used to control for spatial variability in unmeasured factors, thereby increasing the power to detect main effects. Each experimental unit (i.e., a unique combination of AMF and EPN inoculations) consisted of 12 plants arranged in 3 parallel rows of 4, with 1 row per variety (Fig. 1). Within each unit, plants were spaced approximately 10 cm apart, and experimental units were placed at least 0.5 m apart, with plant positions randomized. Blocks were separated by at least 1 m. In total, 240 potato plants were used, corresponding to 10 blocks ×  2 mycorrhizal inoculation treatments × 4 nematode inoculation treatments ×  3plant varieties.

Five weeks after treatment application, we selected plants from four of the ten experimental blocks (*n* = 96 plants) to assess vegetative growth. We measured the total stem height of these plants and counted the number of fully expanded leaves. This same subset was subsequently used to collect constitutive aboveground VOCs and quantify leaf phenolic compounds. The remaining 144 plants from the 6 blocks were manually relocated to a field site adjacent to the greenhouse, where the original experimental design (randomized split-split plot) was maintained for subsequent evaluation of leaf herbivory.

### Volatile organic compound collection

We collected constitutive (i.e., basal levels) aboveground VOCs from plants corresponding to 4 of the 10 blocks (96 plants in total) following the methodology described by Moreira et al. (2024). Briefly, plants were enclosed in 2-L Nalophan bags, and VOCs were trapped on a charcoal filter (SKC sorbent tube filled with Anasorb CSC coconut-shell charcoal) for 2 h using a Sidekick 224-52MTX pump (0.25 L min⁻^1^ airflow of technical air, N₂O₂). Trapped VOCs were then eluted with 150 μL of dichloromethane (CAS#75–09-2; Merck, Dietikon, Switzerland) containing two internal standards (n-octane, CAS#91–20-3, and nonyl acetate, CAS#143–13-5, both at 200 ng in 10 μL dichloromethane). A 1.5 μL aliquot of each extract was injected into an Agilent 7890B gas chromatograph (GC) coupled to a 5977B mass selective detector (MSD) equipped with a 30 m × 0.25 mm × 0.25 μm HP-5MS fused silica column (Agilent, Santa Clara, CA, USA). The GC was operated in pulsed splitless mode (250 °C, injection pressure 15 psi) with helium as the carrier gas. The oven temperature program followed an initial hold at 40 °C for 3.5 min, a ramp of 5 °C min⁻^1^ to 230 °C, and a final hold at 250 °C for 3 min (constant helium flow rate: 0.9 mL min⁻^1^). The transfer line temperature was set to 280 °C. The MSD was operated in electron impact mode (EI) and ionization potential of 70 eV with a scan range of 33–350 (m/z), a source temperature of 230 °C, and a quadrupole temperature of 150 °C.

When available, commercial pure standards were injected to formally identify corresponding volatile compounds. Other identifications were carried out by comparing experimental mass spectra with the references provided by the NIST 17 mass spectral library (US Department of Commerce). In addition, Kováts indices were calculated relative to the retention times of n-alkanes (C8–C20, Sigma-Aldrich, Merck KGaA, Darmstadt, Germany) obtained under identical analytical conditions to support compound identification further. Total VOC emissions were quantified using normalized peak areas and expressed as nanograms per hour (ng h⁻^1^). The normalized peak area for each compound was calculated as the ratio of its integrated peak area to that of the internal standards, adjusting for variation in sample volume during elution (Moreira et al. 2021, 2024; Abdala-Roberts et al. 2022). Reported values thus represent octane-nonyl acetate equivalent nanograms of each compound emitted per plant per hour. Finally, the total VOC emission per sample was obtained by summing the concentrations of individual compounds.

### Phenolic compound quantification

Following the methodology described by Moreira et al. (2024), we quantified constitutive phenolic compounds from the same set of plants used for VOC collection. Three fully expanded leaves per plant were collected, oven-dried at 40 °C for 48 h, and ground using liquid nitrogen to obtain a homogeneous sample. Phenolic compounds were extracted from 20 mg of dry, pulverized leaf tissue using 1 mL of 70% methanol in an ultrasonic bath for 15 min, followed by centrifugation. The extracts were transferred to chromatographic vials and analyzed *via* ultra-high-performance liquid chromatography (UHPLC). Analyses were conducted using a high-performance liquid chromatograph ACQUITY UPLC H-Class PLUS (Waters, Milford, MA, USA) equipped with a photodiode array detector. Separation was performed on a Kinetex™ 2.6 μm C18 100 Å column (100 × 4.6 mm), protected with a C18 guard cartridge. The mobile phase consisted of water-formic acid (0.1%) (solvent A) and acetonitrile-formic acid (0.1%) (solvent B), with a gradient profile of 5% B at injection, increasing to 30% at 4 min, 60% at 106 min, 60% at 25 min, and 100% at 27 min, gradient was returned to initial conditions and stabilized during 2 min before next injection. The flow rate was 0.4 mL min⁻^1^, the column oven temperature was maintained at 25 °C, and the injection volume was 5 μL.

Phenolic compound identification was performed using an ultra-performance liquid chromatography system (Thermo Dionex Ultimate 3000 LC) coupled with electrospray ionization quadrupole time-of-flight mass spectrometry (UPLC-Q-TOF–MS/MS) (Bruker Compact™, Billerica, MA, USA). This approach allowed the identification of two major phenolic groups: flavonoids and caffeic acids. Compound quantification was based on calibration curves generated from standard solutions (0.16, 0.8, 4, 20, 100, and 500 μg mL⁻^1^). We used rutin as the standard for flavonoids and caffeic acid as the standard for hydroxycinnamic acids (Moreira et al. 2018). Phenolic concentrations were expressed as mg g⁻^1^ dry weight tissue.

### Herbivory and mycorrhizal colonization assessments

In June 2024, we assessed herbivory by randomly collecting 10 fully expanded leaves from each of the 144 potato plants relocated in the field. Leaves were selected to be of similar age based on their position along the branch, coloration, and texture. Since plants retained leaves from multiple flushes throughout the growing season, our herbivory estimates represented cumulative damage. The majority of observed damage (over 90% of sampled leaves) was attributed to chewing insects. To quantify leaf herbivory, we photographed each leaf using a Samsung Galaxy A30s (25 MP resolution, 4 × digital zoom) under consistent lighting conditions. The percentage of leaf area consumed by chewing insects was estimated using BioLeaf—Foliar Analysis™, an automated image processing tool for foliar damage quantification (Brandoli Machado et al. 2016). We calculated the mean herbivory percentage per plant for statistical analyses by averaging values across the ten sampled leaves.

To estimate the percentage of root length colonized by AMF, we collected root samples from 12 plants per mycorrhizal treatment. Roots were stained with Sheaffer blue ink, following the protocol outlined by Wilkes et al. (2020), to differentiate AMF structures. The presence of arbuscules and vesicles was quantified using the method described by Trouvelot et al. (1986). All plants, including those in the non-inoculated control group, exhibited a measurable degree of AMF colonization. This outcome is consistent with expectations for field-based experiments, where native AMF communities are naturally present in the soil and capable of colonizing plant roots despite sterilization efforts (Davison et al. 2015). Rather than representing a methodological flaw, the presence of AMF in control plants reflects ecologically realistic conditions under which AMF are widespread and often unavoidable in agricultural systems. Notably, previous studies have shown that commercial AMF inoculants can successfully establish and enhance colonization even in the presence of native fungal communities (Janoušková et al. 2017). In our study, AMF-inoculated plants exhibited significantly higher colonization levels than non-inoculated controls, with an average increase of approximately 30% in both colonization frequency and intensity, with inoculated plants showing greater colonization intensity in root fragments and higher vesicular and arbuscular abundance in the root system (Table S1). Although vesicular and arbuscular abundance in colonized root fragments did not significantly differ between treatments (Table S1), these results confirm that inoculation with *R. irregularis* effectively enhanced mycorrhization at the whole-root level and produced biologically meaningful differences in colonization patterns.

### Statistical analyses

We run linear mixed models (LMMs) to assess the effects of mycorrhizal inoculation (two levels: control and mycorrhization), nematode inoculation (four levels: no larvae, dead larvae, and dead larvae infected with *H. bacteriophora* or *S. carpocapsae*), and their interactions on plant growth (total stem height, number of leaves) and direct defenses (phenolic compounds). Response variables were log-transformed to ensure the normality of residuals. For indirect defenses (total VOC emissions) and leaf-chewing herbivory (percentage of leaf area consumed), we applied generalized linear mixed models (GLMMs) with a zero-inflated gamma (ziGamma) distribution (logit link function). Plant genotype and block were included as random factors in all models to account for potential genetic variation in resistance or trait expression across cultivars and spatial effects. Treating cultivar as a random effect allowed us to generalize our findings across a broader range of genotypes while maintaining statistical power. However, to explore cultivar-specific responses, we also conducted complementary analyses treating cultivar as a fixed effect. These analyses revealed significant differences among cultivars for several traits (see Table S2 and Fig. S1), highlighting the presence of genotype-level variation. Notably, the main treatment effects and their interactions remained qualitatively consistent across modeling approaches, supporting the robustness of our findings and their generalizability beyond the specific cultivars tested. All statistical analyses were performed in *R* ver. 4.3.0 (R Core Team 2023), using the *glmer* function from the *lme4* package to conduct GLMMs (Bates et al. 2015). We obtained model-derived least-square means and associated standard errors, which were back-transformed for interpretability in the case of LMMs and GLMMs. When significant treatment effects were detected, we conducted post hoc pairwise comparisons using Tukey-adjusted means via the lsmeans function from the *lsmeans R* package (Lenth 2016).

To further analyze VOCs, we conducted follow-up tests on individual compounds, applying false discovery rate (FDR) adjustments for *P* < 0.05 (Benjamini and Hochberg 1995) to minimize Type I error due to multiple comparisons. In addition, we used permutational multivariate analysis of variance (PERMANOVA) to test the effects of mycorrhizal and nematode inoculations and their interaction on VOC composition. This analysis was based on compound abundances and 10,000 permutations, implemented using the *vegan R* package (Oksanen et al. 2016). To visualize the PERMANOVA results, we performed a principal coordinates analysis (PCoA) based on Bray–Curtis pairwise dissimilarities and plotted the centroids of each mycorrhizal and nematode treatment separately (Moreira et al. 2021). In addition, we identified VOCs that strongly correlated (R^2^ > 0.7) with the first two ordination axes using the *envfit* function in *vegan R* package and represented these relationships as biplot arrows scaled to R^2^ values.

## Results

### Plant growth

**Fig. 2:**
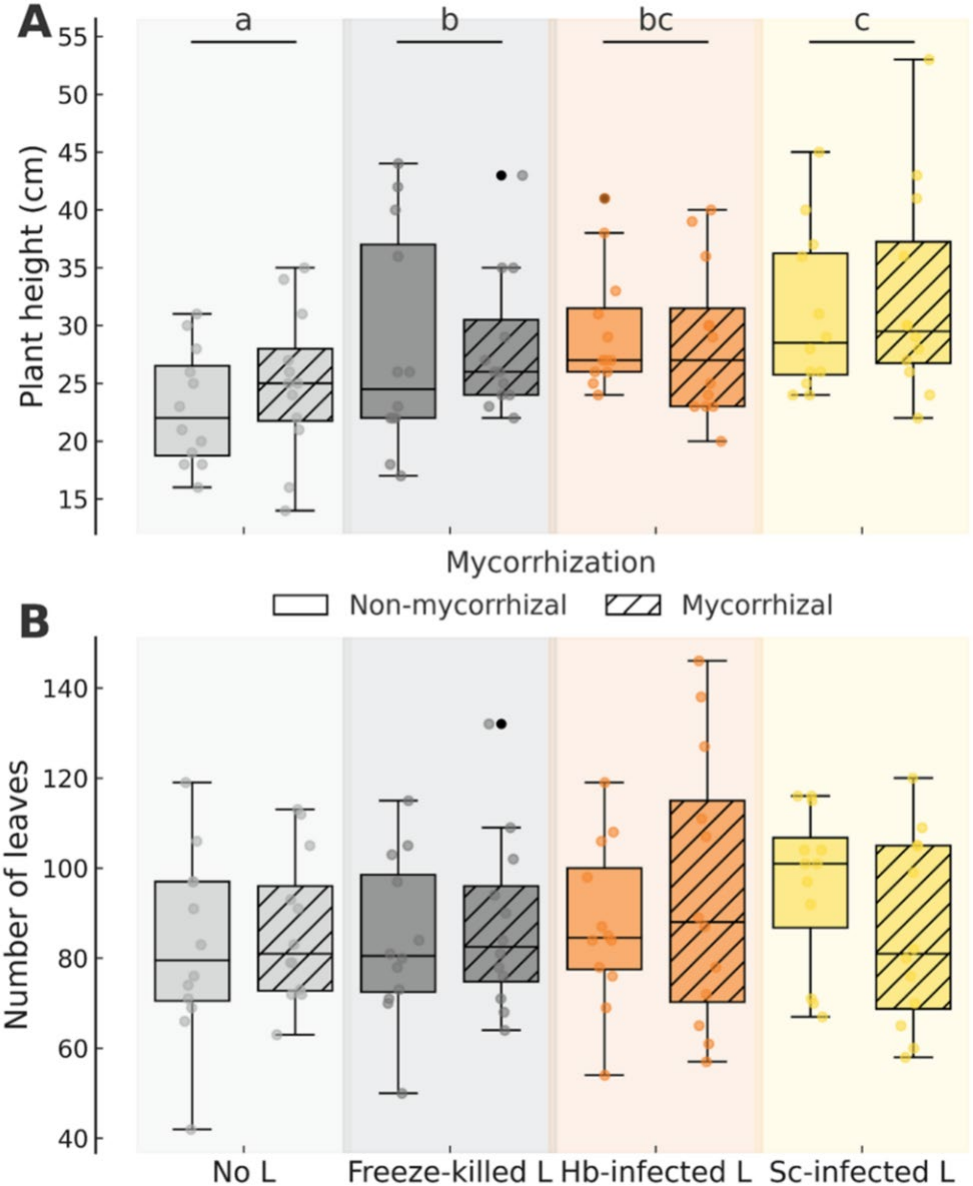
**A**, **B** Effects of mycorrhizal (two levels: mycorrhization by *Rhizoglomus irregulare* vs. no mycorrhization) and nematode (four levels: no larvae [L], freeze-killed L, and *Heterorhabditis bacteriophora* [Hb] or *Steinernema carpocapsae* [Sc] infected L) inoculations on height (cm) (**A**) and number of leaves (**B**) in potato (*Solanum tuberosum*) plants. Letters indicate statistically significant differences (*P* < 0.05) based on Tukey's post-hoc tests from linear mixed models among nematode treatments. No significant differences were found for pairwise comparison between mycorrhizal and non-mycorrhizal plants within nematode treatments. Statistical details are provided in Table 2

Mycorrhization had no significant overall effect on plant height or number of leaves, and no interaction was detected between mycorrhization and nematode inoculation for either trait (Table 2, Fig. 2). In contrast, nematode inoculations significantly affected plant height (Table 2) but had no effect on leaf number. Post hoc comparisons revealed that plants in the no-larvae treatment were significantly shorter than those exposed to freeze-killed larvae (19.6% increase, *P* = 0.007), larvae infected with *H. bacteriophora* (15.2% increase, *P* < 0.001), or *S. carpocapsae* (30.5% increase, *P* < 0.001; Fig. 2A). In addition, plants treated with *S. carpocapsae*-infected larvae grew on average 9.1% taller than those exposed to freeze-killed larvae (*P* = 0.049; Fig. 2A).

**Table 2:** Results from mixed models testing the effects of mycorrhizal inoculation (two levels: control and mycorrhization), nematode inoculation (four levels: no larvae, freeze-killed larvae, and infected larvae with *Heterorhabditis bacteriophora* or *Steinernema carpocapsae*), and their interaction (all fixed factors) on plant growth (height, number of leaves), total emission and composition of volatile organic compounds (VOCs), the concentration of leaf phenolic compounds (flavonoids, and hydroxycinnamic acids) (*n* = 96), and leaf herbivory (%) (*n* = 144) by chewing insects in potato (*Solanum tuberosum*) plants

Response	Mycorrhizal inoculation (M)			Nematode inoculation (N)			M x N		
	DF	χ^2^/F/pseudo-F	*P*	DF	χ^2^/F/pseudo-F	*P*	DF	χ^2^/F/pseudo-F	*P*
Plant height	1, 83	0.58	0.445	3, 83	37.60	** < 0.001**	3, 83	1.76	0.623
Number of leaves	1, 83	0.03	0.860	3, 83	2.02	0.569	3, 83	3.01	0.390
Total VOC emission	1, 84	3.43	0.064	3, 84	10.74	**0.013**	3, 84	8.13	**0.043**
VOC composition	1, 83	0.55	0.642	3, 83	0.83	0.545	3, 83	0.58	0.804
Flavonoids	1, 83	0.04	0.840	3, 83	11.06	**0.011**	3, 83	0.98	0.807
Hydroxycinnamic acids	1, 83	0.38	0.538	3, 83	3.49	0.322	3, 83	4.61	0.203
Leaf herbivory (%)	1, 124	2.05	0.152	3, 124	4.39	0.220	3, 124	9.46	**0.024**

For total VOC emission and leaf herbivory, we used a generalized linear mixed model (ziGamma distribution), while VOC composition was analyzed using a permutational multivariate analysis of variance (PERMANOVA). For the rest of the variables we used a linear mixed model. The table reports degrees of freedom (DF; numerator, denominator), χ^2^ (for total VOCs and leaf herbivory), Pseudo-F values (for VOC composition), or F-values (for plant height, number of leaves, flavonoids and hydroxycinnamic acids) along with their significance levels (*P*). Significant *P*-values (*P* < 0.05) are highlighted in bold

### Volatile organic compound emissions

**Fig. 3:**
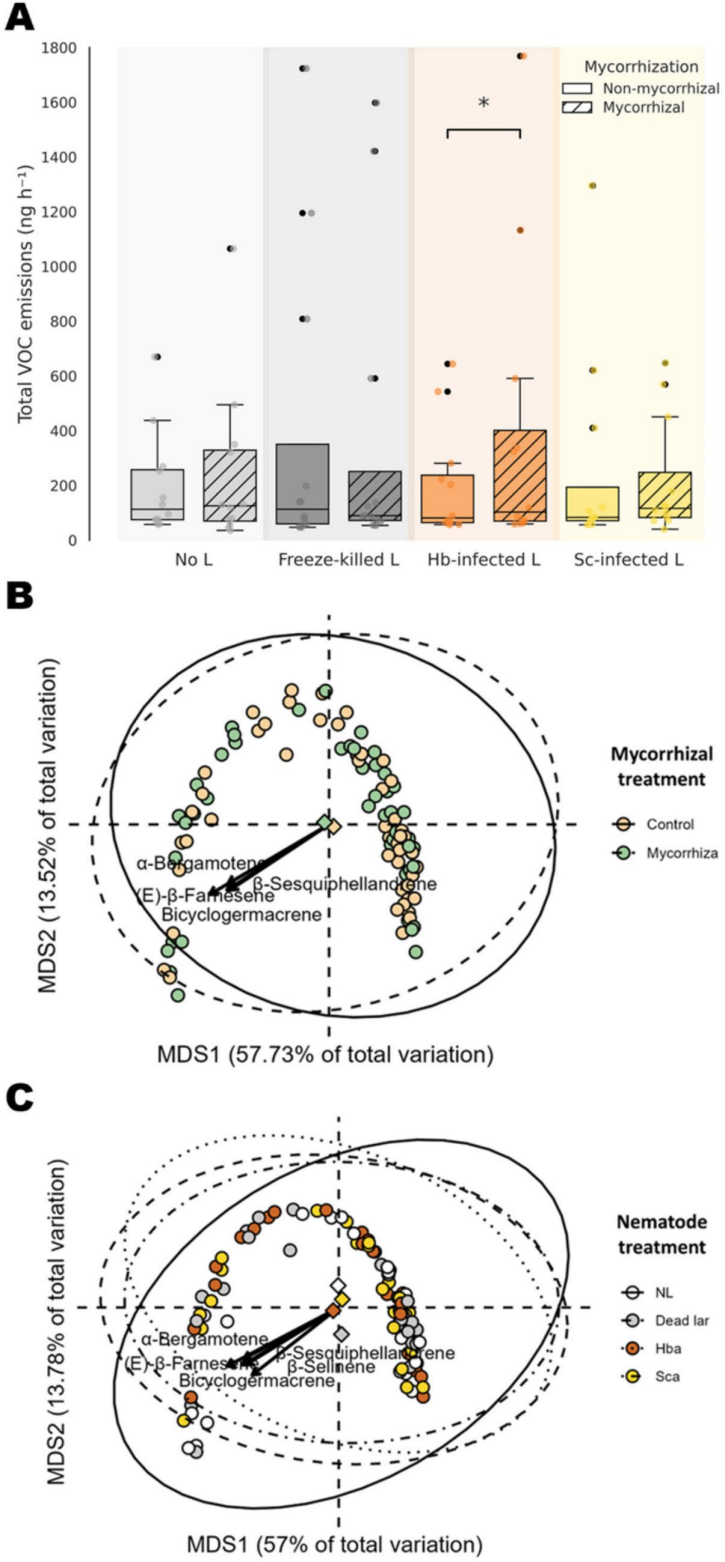
**A** Effects of mycorrhizal (two levels: mycorrhization by *Rhizoglomus irregulare* vs. no mycorrhization) and nematode (four levels: no larvae [L], freeze-killed L, and *Heterorhabditis bacteriophora* [Hb] or *Steinernema carpocapsae* [Sc] infected L) inoculations on the total emission of volatile organic compounds (VOCs) in potato (*Solanum tuberosum*) plants. Emissions are expressed as normalized peak areas (ng h⁻^1^). Asterisks indicate statistically significant differences (*P* < 0.05) based on pairwise comparisons between mycorrhizal and non-mycorrhizal plants within each nematode treatment using linear mixed-effects models. **B**, **C** Unconstrained ordination plots (Principal Coordinates Analysis, PCoA) illustrate the effects of mycorrhizal (**B**) and nematode (**C**) treatments on VOC composition. Biplot vectors indicate the most influential VOCs, with arrow lengths scaled according to their *R*^*2*^ values to reflect relative contribution to ordination axes. Each diamond represents the centroid of a treatment group, with associated 95% confidence ellipses. The first two axes captured 71.25% and 70.78% of the total variation in VOC composition explained by mycorrhizal and nematode treatments, respectively. See Table 2 for statistical details

Mycorrhization had no significant overall effect on total VOC emissions from potato leaves (Table 2, Fig. 3A). However, in plants inoculated with *H. bacteriophora*, mycorrhizal individuals emitted significantly more VOCs than non-mycorrhizal ones (98.6% increase; *p* = 0.043), with a marginally significant increase also observed in the no-larvae treatment (23.3% increase; *P* = 0.064; Fig. 3A). In contrast, nematode inoculations significantly influenced total VOC emissions, although post hoc comparisons did not reveal statistically significant differences among nematode treatments. A significant interaction between nematode and mycorrhizal inoculations was also detected (Table 2). Regarding VOC composition, PERMANOVA detected no significant effects of mycorrhizal or nematode inoculations or their interaction on overall compound profiles (Table 2). However, follow-up analyses revealed significant differences in the emission of individual compounds (see Table S3). Specifically, we found that seven compounds (e.g., α-copaene, β-elemene, and β-selinene) showed significant differences in emissions under the mycorrhizal treatment. Ten compounds (e.g., β-elemene, Bicyclogermacrene, and β-sesquiphellandrene) exhibited significant differences under the nematode treatment. Furthermore, eight compounds (e.g., 1,3,7-nonatriene, 4,8-dimethyl-, (3E)- [also known as DMNT], β-elemene, and (E)-β-farnesene) showed significant differences in emissions under the interaction between both treatments.

### Leaf phenolic compounds

**Fig. 4:**
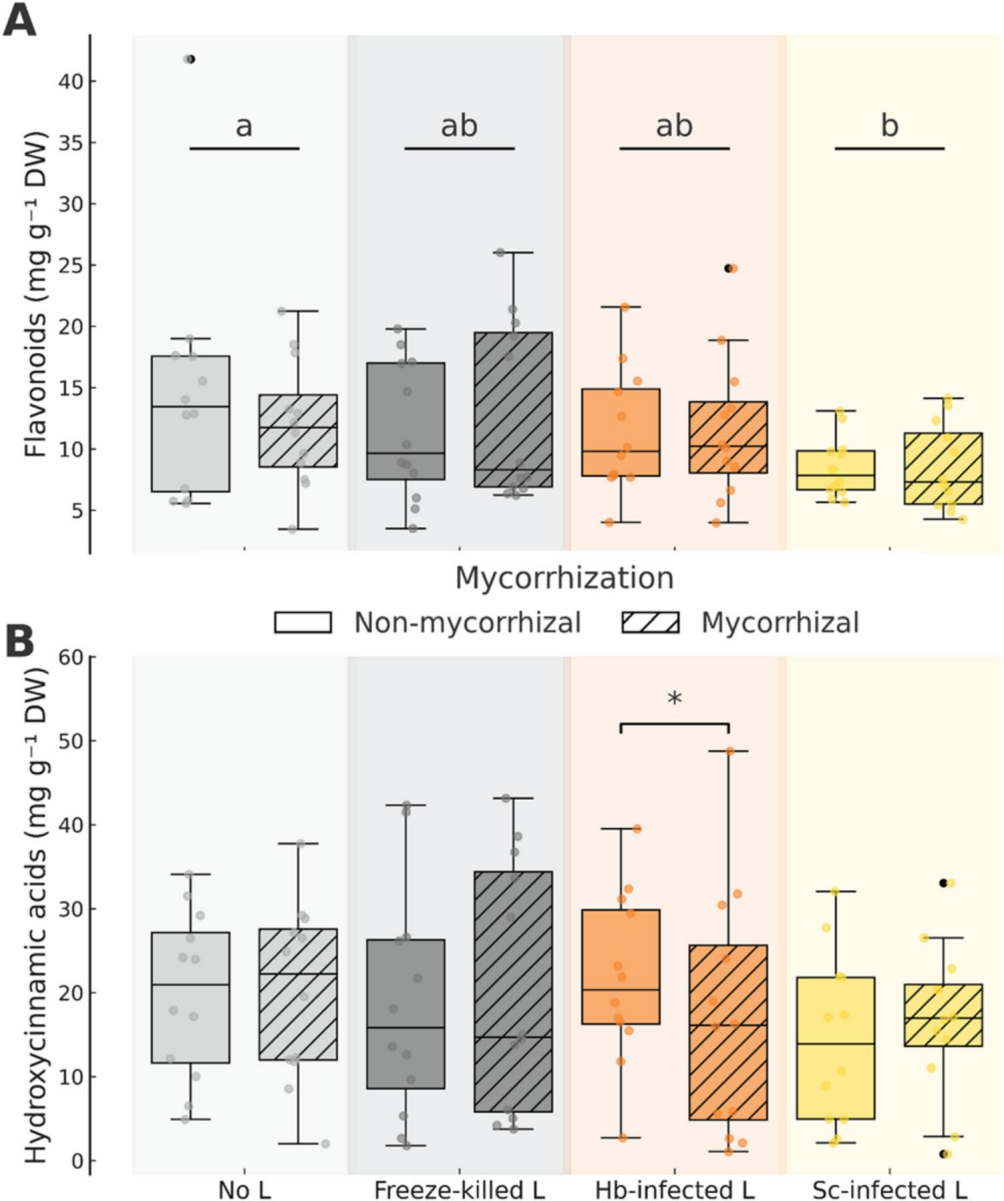
**A, B** Effects of mycorrhizal (two levels: mycorrhization by *Rhizoglomus irregulare* vs. no mycorrhization) and nematode (four levels: no larvae [L], freeze-killed L, and *Heterorhabditis bacteriophora* [Hb] or *Steinernema carpocapsae* [Sc] infected L) inoculations on flavonoids (**A**) and hydroxycinnamic acids (**B**) in potato (*Solanum tuberosum*) plants. Letters and asterisks indicate statistically significant differences (*P* < 0.05) from linear mixed-effects models based on Tukey-adjusted post hoc comparisons among nematode treatments and pairwise differences between mycorrhizal and non-mycorrhizal plants within nematode treatments, respectively. Statistical details are provided in Table 2

Mycorrhization had no significant overall effect on flavonoid or hydroxycinnamic acid contents in potato leaves, and no interaction was detected between mycorrhization and nematode inoculation for either trait (Table 2, Fig. 4). However, in plants inoculated with *H. bacteriophora*, mycorrhizal individuals accumulated significantly less hydroxycinnamic acids than non-mycorrhizal ones (20.6% decrease; *P* = 0.039; Fig. 4B). Nematode inoculations did not affect hydroxycinnamic acid contents, but did significantly influence flavonoid concentrations (Table 2). Post hoc comparisons showed that plants inoculated with *S. carpocapsae*-infected larvae accumulated significantly less flavonoids than those without larval inoculation (24.7% decrease; *P* = 0.012), with a marginally significant decrease also observed following freeze-killed larval application (8.2% decrease; *P* = 0.080; Fig. 4A).

### Leaf herbivory

**Fig. 5:**
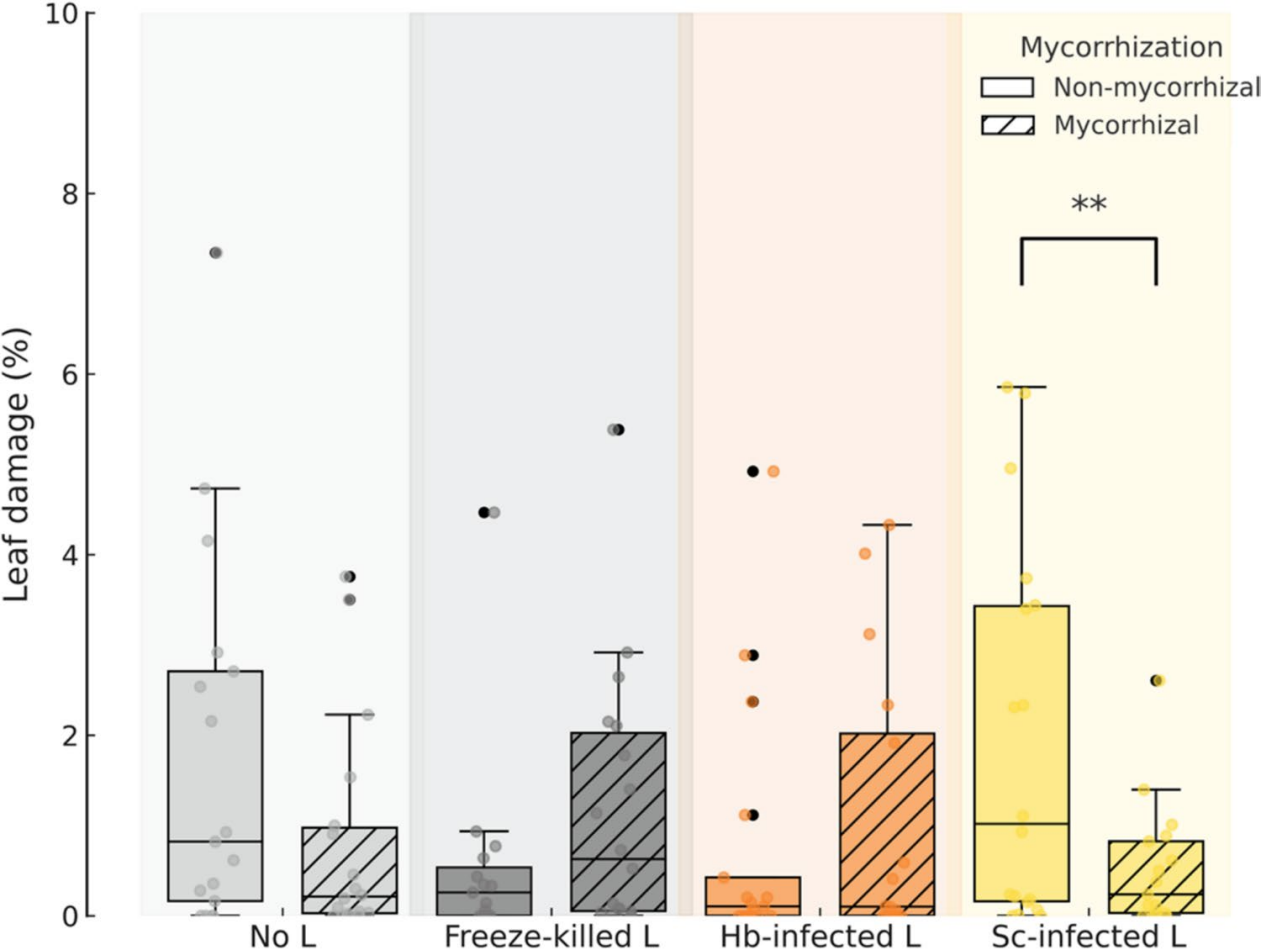
Effects of mycorrhizal (two levels: mycorrhization by *Rhizoglomus irregulare* vs. no mycorrhization) and nematode (four levels: no larvae [L], freeze-killed L, and *Heterorhabditis bacteriophora* [Hb] or *Steinernema carpocapsae* [Sc] infected L) inoculations on the percentage of leaf area consumed by chewing insects in potato (*Solanum tuberosum*) plants. Asterisks indicate statistically significant differences (*P* < 0.01) based on pairwise comparisons between mycorrhizal and non-mycorrhizal plants within each nematode treatment using linear mixed-effects models. Statistical details are provided in Table 2

While neither mycorrhizal nor nematode treatments individually affected chewing herbivory, a significant interaction between the two factors was detected (Table 2). In plants inoculated with *S. carpocapsae*, mycorrhizal individuals experienced significantly greater leaf damage than non-mycorrhizal ones (79.5% increase; *P* = 0.007), with a marginally significant decrease also observed in the no-larvae treatment (23.3% decrease; *P* = 0.074; Fig. 5).

## Discussion

Plant-associated mutualists frequently coexist and interact in agroecosystems, yet the ecological and functional outcomes of these multi-partner associations remain poorly understood. In this study, using potato (*S. tuberosum*) plants as a model crop, we examined how two ecologically distinct belowground symbionts—EPNs and AMF—influence plant phenotype and aboveground herbivore interactions via plant-mediated mechanisms. Our results showed that EPNs alone significantly enhanced plant growth and altered foliar flavonoid concentrations, consistent with previous findings that suggest EPNs modulate plant physiology via systemic signaling triggered by infected cadavers (Helms et al. 2019). Surprisingly, AMF inoculation did not produce consistent main effects on plant growth, chemical defenses, or herbivory. However, AMF influenced specific plant traits in a context-dependent manner, contingent on the identity of the co-occurring EPN species. For instance, in mycorrhizal plants exposed to *H. bacteriophora*, we observed increased VOC emissions and reduced levels of hydroxycinnamic acids on leaves, potentially indicating a shift toward indirect defense signaling at the expense of direct chemical defenses. In contrast, mycorrhization in combination with *S. carpocapsae* was associated with decreased levels of herbivore damage. These findings partially support our initial hypothesis and underscore that the combined effects of belowground mutualists on aboveground plant traits are highly dependent on the specific symbiont pairing. This specificity is ecologically relevant, as it suggests that particular combinations of root-associated mutualists can differentially shape plant responses even within the diverse microbial communities expected of complex agroecosystems.

In the current study, *R. irregularis* successfully colonized potato roots at the structural level. Mycorrhizal plants exhibited significantly greater colonization frequency and intensity, as well as greater vesicle and arbuscular abundance across the whole root system—patterns consistent with previous reports in potato and other crops (Buysens et al. 2017; Moreira et al. 2024). Despite this successful colonization, AMF inoculation did not significantly affect any of the measured plant traits, contrasting with earlier findings that showed *R. irregularis* promoting biomass accumulation and enhancing chemical defenses under specific environmental contexts (Buysens et al. 2017; Schoenherr et al. 2019; Moreira et al. 2024). Notably, using a similar experimental setup, Moreira et al. (2024) reported increased total VOC emissions in mycorrhizal potato plants, suggesting functional shifts in indirect defense. A likely explanation for the absence of effects in our study is the relatively benign environmental conditions under which the experiment was conducted. The benefits of AMF often become more apparent under abiotic or biotic stress, such as drought, nutrient limitation, or pathogen attack (van der Heijden et al. 1998; Begum et al. 2019). In the absence of such constraints, plants may operate near their physiological optimum, reducing their reliance on mycorrhizal partners and thereby limiting the functional expression of the symbiosis (Smith and Smith 2011).

In contrast to the limited impact of AMF, treatments involving insect cadavers—either freeze-killed or infected with EPNs—exerted measurable effects on plant phenotype. Nematode-associated cadavers, in particular, significantly increased plant height, a response consistent with previous studies showing that EPN-related cues can modulate plant physiology, including shoot growth stimulation (Wang et al. 2024). Most prior evidence of EPN-induced growth promotion involves potato plants subjected to herbivore or pathogen attack, including plant-parasitic nematodes (e.g., El Aimani et al. 2022), suggesting that the observed height increase likely arose from direct physiological responses to EPN-associated signals rather than the alleviation of biotic stress. This stimulation is thought to result from systemic signaling triggered by compounds released from infected cadavers, such as microbial elicitors or modified root exudates, which may influence hormone regulation or nutrient dynamics (Jagdale and Grewal 2008; Helms et al. 2019). In addition to growth effects, *S. carpocapsae*-infected cadavers exhibited reduced foliar flavonoid concentrations, a pattern also observed in other systems, consistent with reports that EPNs or their symbiotic partners can downregulate plant secondary metabolite production (Wang et al. 2024). In contrast, *H. bacteriophora* had no significant effect on flavonoid levels, suggesting species-specific differences in the modulation of plant defense chemistry, which their respective microbial symbionts may mediate. Although post hoc comparisons among nematode treatments did not reveal statistically significant pairwise differences in VOC emissions, the overall model indicated a significant treatment effect, pointing to subtle changes in indirect defense signaling (Helms et al. 2019). This pattern may reflect a physiological shift in resource allocation away from constitutive chemical defenses toward growth, in line with a compensatory response to belowground cues originating from EPN-infected cadavers (Züst and Agrawal 2017; Wang et al. 2024). While empirical evidence linking EPNs to changes in plant VOC emissions remains limited, our findings add to growing research suggesting that aboveground volatile responses can be influenced by belowground signals associated with EPN activity (Helms et al. 2019; Wang et al. 2024).

The interaction between AMF and EPN-infected cadavers produced trait-specific outcomes that were not predictable based on their individual effects, underscoring the context-dependent nature of multi-mutualist associations. In the case of *H. bacteriophora*, mycorrhization intensified plant volatile emissions, suggesting that the co-occurrence of these symbionts may enhance certain indirect defenses via converging signaling pathways. Similar modulation has been observed in other systems where mutualists interact through shared hormonal or metabolic pathways (e.g., Fernández et al. 2014; Helms et al. 2019). In our study, we observed a particularly intriguing pattern in VOC emissions. Several individual compounds—including terpenes such as DMNT, (E)-β-farnesene, and α-bergamotene, previously reported as ecologically relevant in potato plants and typically associated with herbivore-induced responses (e.g., Vázquez-González et al. 2022; Martín-Cacheda et al. 2023)—were emitted in greater amounts by plants exposed to non-infected freeze-killed larvae. Although previous studies have shown that EPN-infected cadavers can alter plant VOC profiles (e.g., Zhang et al. 2019; Wang et al. 2024), no prior work has demonstrated that insect cadavers alone can elicit such changes in the absence of herbivory or EPN infection. Moreover, this effect appeared to be attenuated in the presence of AMF, which are known to influence microbial saprotrophic activity and reshape soil microbial communities (Hodge et al. 2001; Gui et al. 2017). These changes can alter the decomposition of organic matter, including insect cadavers, potentially affecting the accumulation or persistence of decomposition-related VOCs that mediate plant responses. In contrast, the combination of AMF and *S. carpocapsae*-infected cadavers led to a significant reduction in herbivore damage, a response not observed in either single-inoculation treatment. While AMF and EPNs have independently been shown to influence plant defense traits (Schoenherr et al. 2019; Locke and Crawford 2022; Wang et al. 2024), this may represent one of the first documented cases in which aboveground herbivory is mitigated through a context-dependent interaction between two root-associated mutualists. Although some studies have evaluated combinations of beneficial soil organisms, including EPNs, *Pseudomonas* bacteria, and mycorrhizae, they have not found apparent synergistic or additive effects on plant defense outcomes (Imperiali et al. 2017; Jaffuel et al. 2019). In contrast, our results suggest a functional, partner-specific interaction between AMF and EPNs, as evidenced by the combination of *R. irregularis* and *S. carpocapsae*-infected cadavers leading to measurable reductions in herbivore damage, an outcome not observed in either single-inoculation treatment. These findings suggest a mutualist-specific integration of belowground cues, wherein the identity of the EPN partner determines whether the interaction amplifies or modulates plant defense expression.

The observed species-specificity highlights how subtle differences between symbionts can produce divergent outcomes across plant traits. Importantly, the combined effects were not uniformly beneficial, emphasizing that multi-partner symbioses do not necessarily align to optimize host performance. Similar patterns of conditional non-additivity have been reported in other systems (Tsiknia et al. 2021; Moreira et al. 2024), reinforcing the need to move beyond binary models of symbiosis and toward a more nuanced framework that accounts for partner compatibility, functional complementarity, and environmental context. While mutualistic outcomes are often expected in AMF–plant interactions, especially under agricultural conditions, accumulating evidence shows that the strength and direction of these associations can vary depending on host genotype, environmental factors, and the identity of the fungal strain (Johnson et al. 1997; Werner and Kiers 2015). In our study, inoculation with *R. irregularis* led to a significant increase in root colonization compared to non-inoculated controls, confirming the biological activity of the inoculant under realistic field conditions. Rather than undermining the concept of mutualism, the observed variability highlights its ecological complexity and underscores the importance of context-dependent interactions in shaping plant phenotype. Embracing this complexity is essential for advancing our understanding of multi-partner symbioses and for developing more predictive frameworks in sustainable agriculture.

This study highlights the complexity and context dependency of multi-mutualist interactions, revealing that specific pairings of belowground symbionts can modulate aboveground plant traits in non-additive ways. To build on these findings, future research should explore whether such partner-specific effects persist under more ecologically realistic conditions. In particular, testing mixed AMF communities —rather than single strains—would better reflect the diversity typically found in agroecosystems and help assess the robustness of observed interactions. In addition, further mechanistic studies are needed to uncover the physiological and molecular bases of these interactions. Investigating the role of hormonal cross-talk (e.g., jasmonic acid, salicylic acid, and ethylene pathways), microbial signaling compounds, and host gene expression will be critical to understanding how plants integrate multiple belowground cues. Further, while our results suggest shifts in VOC emissions, their ecological relevance remains unclear. Future work should assess how specific VOC changes influence herbivore behavior or multitrophic interactions, ideally under field conditions where environmental variability and microbial complexity may amplify or dampen mutualistic effects.

## Conclusion

Our study reveals that interactions between functionally distinct belowground mutualists can modulate plant growth and aboveground defense traits in a context-dependent manner. While AMF alone did not consistently alter plant phenotype, their presence modified the effects of EPNs on volatile emissions and herbivory, highlighting the importance of symbiont identity and compatibility. Notably, we found that insect cadavers alone, even in the absence of EPN infection, can trigger the emission of ecologically relevant volatiles, and that this response is attenuated by AMF, likely through changes in decomposition dynamics. These findings underscore the complexity of multitrophic interactions in the rhizosphere and suggest that integrating multiple mutualists may offer novel strategies for enhancing crop resilience. Future research should explore the mechanistic basis of these interactions and their ecological relevance under field conditions.

## Supplementary Information

Below is the link to the electronic supplementary material.

Supplementary file1 (DOCX 2439 KB)

## Data Availability

The data presented in this study will be archived at https://digital.csic.es/, ensuring compliance with the FAIR mandate and accessibility to all researchers.

## Abbreviations

AMF: Arbuscular mycorrhizal fungi
EPN: Entomopathogenic nematode
VOC: Volatile organic compound
