# Supplementary material for: Belowground mutualists modulate growth and aboveground defense in potato: insights from mycorrhizal and entomopathogenic nematode interactions
Source: Planta. 2025 Nov 25;263(1):15. doi: 10.1007/s00425-025-04877-w (PMC12644124; doi:10.1007/s00425-025-04877-w)
Supplement: Supplementary file 1 — Supplementary file1 (DOCX 2439 KB) [file 425_2025_4877_MOESM1_ESM.docx]

**SUPPLEMENTARY MATERIAL**

**Table S1** Parameters analysed to quantify AMF colonization of potato plants under mycorrhizal treatment with two levels: no mycorrhization (control) vs. mycorrhization by *Rhizoglomus irregulare*. Means (± SE) for each treatment, degrees of freedom, t-student values, and associated significance levels (*p*) are shown. Significant *P* values (*P* < 0.05) are highlighted in bold.

|  | **Control** | **Mycorrhization** | **T-student** | **Df** | ***p*** |
| --- | --- | --- | --- | --- | --- |
| **F%** | 87.5±4.46 | 100±0 | 2.80 | 11.00 | **0.02** |
| **M%** | 17.1±3.48 | 53.92±4.76 | 6.31 | 20.17 | **<0.001** |
| **m%** | 18.36±3.38 | 53.92±4.76 | 6.15 | 19.86 | **<0.001** |
| **v%** | 51.89±6.74 | 44.9±4.97 | -0.83 | 20.37 | 0.41 |
| **V%** | 10.38±2.20 | 24.41±4.09 | 3.09 | 17 | **0.006** |
| **a%** | 48.13±5.21 | 35.64±4.65 | -1.77 | 21.73 | 0.08 |
| **A%** | 9.7±2.24 | 18.34±2.71 | 2.43 | 21.13 | **0.02** |

F%: frequency of mycorrhization in the root system.

M%: intensity of the mycorrhizal colonization in the root system.

m%: intensity of the mycorrhizal colonization in the root fragments.

v%: vesicular abundance in mycorrhizal parts of root fragments.

V%: vesicular abundance in the root system.

a%: arbuscular abundance in mycorrhizal parts of root fragments.

%: arbuscular abundance in the root system


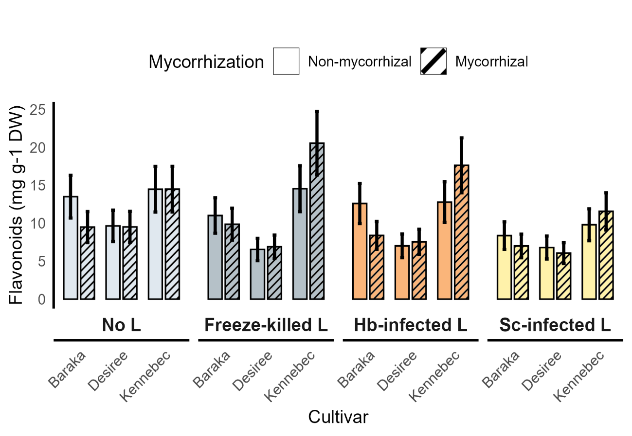

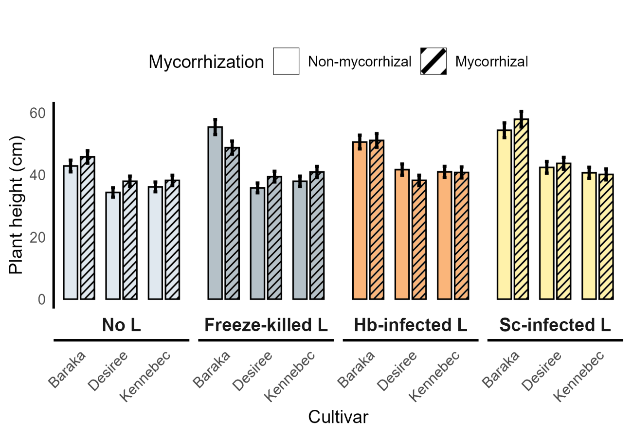

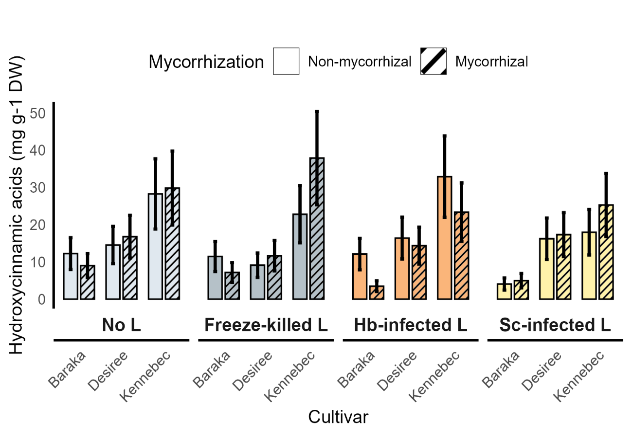

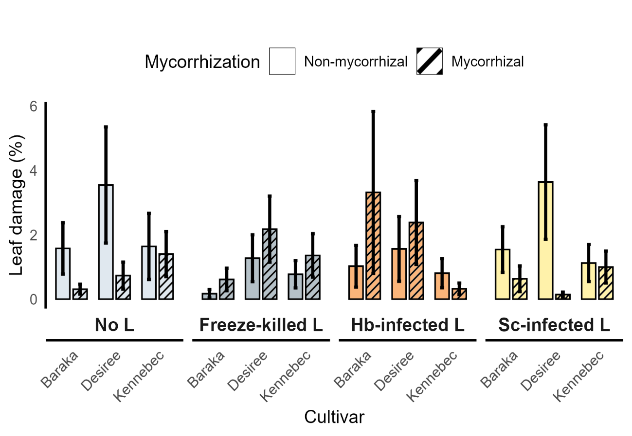

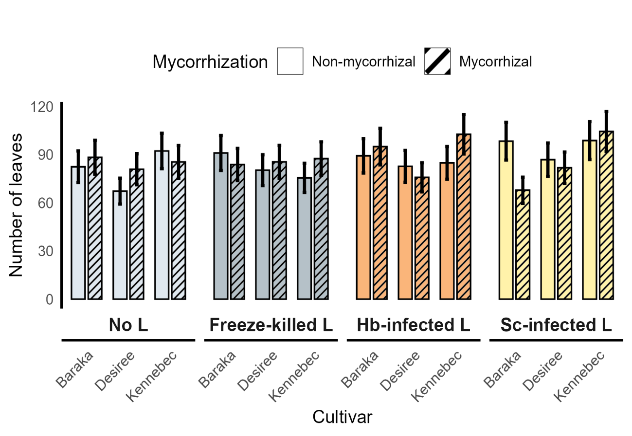

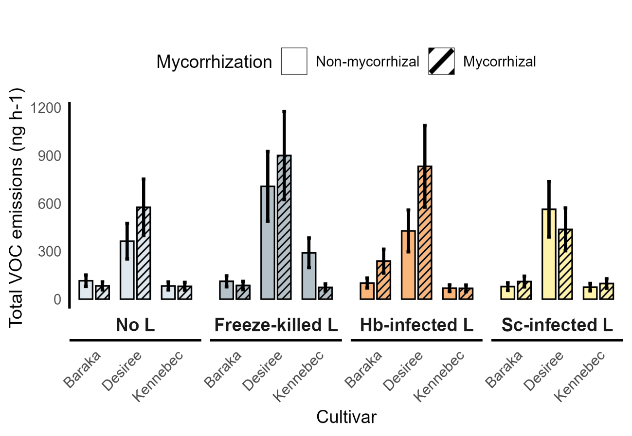


**A**

**B**

**C**

**D**

**E**

**F**


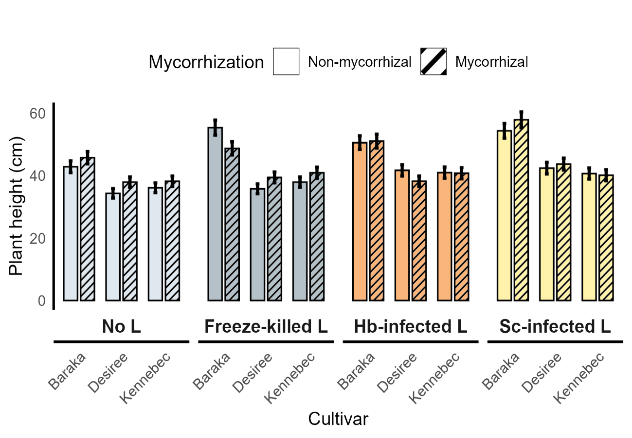


**Fig. S1**. Effects of mycorrhizal (two levels: mycorrhization by *Rhizoglomus irregulare vs*. no mycorrhization) and nematode (four levels: no larvae [L], freeze-killed L, and *Heterorhabditis bacteriophora* [Hb] or *Steinernema carpocapsae* [Sc] infected L) inoculations in three potato (*Solanum tuberosum*) cultivars on (**A**) plant height, (**B**) number of leaves, (**C**) flavonoids and (**D**) hydroxycinnamic acids, (**E**) total aboveground emissions of volatile organic compound (VOC), and (**F**) leaf damage. Statistical details are provided in Table S2.

**Table S2**. Results from mixed models testing the effects of mycorrhizal inoculation (two levels: control and mycorrhization), nematode inoculation (four levels: no larvae, freeze-killed larvae, and infected larvae with Heterorhabditis bacteriophora or Steinernema carpocapsae), plant cultivar (three levels: Kennebec, Baraka, and Desiree), and their interaction (all fixed factors) on plant growth (height, number of leaves), total emission and composition of volatile organic compounds (VOCs), the concentration of leaf phenolic compounds (flavonoids, and hydroxycinnamic acids), and leaf herbivory (%) by chewing insects in potato (Solanum tuberosum) plants.

|  |  | **Plant** | **Number** | **Total VOC** | **VOC** | **Flavonoids** | **Hydroxycinnamic** | **Leaf herbivory** |
| --- | --- | --- | --- | --- | --- | --- | --- | --- |
|  |  | **height** | **of leaves** | **emission** | **composition** |  | **acids** | **(%)** |
| **Mycorrhizal** | df | 1, 72 | 1, 69 | 1, 70 | 1, 71 | 1, 69 | 1, 69 | 1, 109 |
| **inoculation (M)** | χ2/F/pseudo-F | 1.54 | 0.03 | 0.42 | 0.46 | 0.04 | 0.38 | 1.46 |
|  | *p* | 0.218 | 0.861 | 0.518 | 0.661 | 0.843 | 0.539 | 0.227 |
| **Nematode** | df | 3, 72 | 3, 69 | 3, 70 | 3, 72 | 3, 69 | 3, 69 | 3, 109 |
| **inoculation (N)** | χ2/F/pseudo-F | 15.09 | 0.67 | 1.04 | 0.71 | 3.59 | 1.16 | 7.09 |
|  | *p* | **<0.001** | 0.573 | 0.791 | 0.621 | **0.019** | 0.329 | 0.069 |
| **Plant** | df | 2, 72 | 2, 69 | 2, 70 | 2, 72 | 2, 69 | 2, 69 | 2, 109 |
| **Cultivar (C)** | χ2/F/pseudo-F | 90.98 | 2.858 | 19.61 | 28.64 | 21.09 | 29.46 | 9.69 |
|  | *p* | **<0.001** | 0.064 | **< 0.001** | **< 0.001** | **< 0.001** | **< 0.001** | **0.007** |
|  | df | 3, 72 | 3, 69 | 3, 70 | 3, 72 | 3, 69 | 3, 69 | 3, 109 |
| **M x N** | χ2/F/pseudo-F | 1.37 | 1 | 5.37 | 0.49 | 0.31 | 1.54 | 9.61 |
|  | *p* | 0.258 | 0.398 | 0.147 | 0.831 | 0.815 | 0.212 | **0.022** |
|  | df | 2, 72 | 2, 69 | 2, 70 | 2, 72 | 2, 69 | 2, 69 | 2, 109 |
| **M x C** | χ2/F/pseudo-F | 0.31 | 1.11 | 7.59 | 0.78 | 2.83 | 2.10 | 3.17 |
|  | *p* | 0.734 | 0.334 | **0.022** | 0.503 | 0.065 | 0.129 | 0.205 |
|  | df | 6, 72 | 6, 69 | 6, 70 | 6, 72 | 6, 69 | 6, 69 | 6, 109 |
| **N x C** | χ2/F/pseudo-F | 1.65 | 0.91 | 8.36 | 0.58 | 0.69 | 1.35 | 26.58 |
|  | *p* | 0.145 | 0.497 | 0.213 | 0.861 | 0.658 | 0.248 | **< 0.001** |
|  | df | 6, 72 | 6, 69 | 6, 70 | 6, 72 | 6, 69 | 6, 69 | 6, 109 |
| **M x N x C** | χ2/F/pseudo-F | 1.67 | 1.01 | 9.08 | 0.59 | 0.18 | 0.31 | 13.77 |
|  | *p* | 0.141 | 0.423 | 0.169 | 0.839 | 0.981 | 0.932 | **0.032** |

For total VOC emission and leaf herbivory, we used a generalized linear mixed model (ziGamma distribution), while VOC composition was analyzed using a permutational multivariate analysis of variance (PERMANOVA). For the rest of the variables we used a linear mixed model. The table reports degrees of freedom (DF; numerator, denominator), χ² (for total VOCs and leaf herbivory), Pseudo-F values (for VOC composition), or F-values (for plant height, number of leaves, flavonoids and hydroxycinnamic acids) along with their significance levels (*p*). Significant p-values (*p* < 0.05) are highlighted in bold.

**Table S3.** Means (± SE) for emission of individual volatile organic compounds (VOCs) (octane and nonyl acetate-equivalent ng h^-1^), identified by GC-MS, by potato (*Solanum tuberosum*) plants under four different nematode treatments (Nem): no larvae, freeze-killed larvae, and infected larvae with *Heterorhabditis bacteriophora* (Hba) or *Steinernema carpocapsae* (Sca), and under two mycorrhizal treatments (Myc) (control *vs*. mycorrhization by *Rhizoglomus irregulare*)^1^ (N=12). Significance le

vels (*p*) are shown, with compounds exhibiting a significant treatment effect (*p* < 0.05) highlighted in bold. Different letters indicate statistically significant differences (*p* < 0.05) based on Tukey's post-hoc tests from generalized linear mixed models for those individual VOCs that showed significant differences in emissions under the interaction between mycorrhizal and nematode treatments.

^1^We performed *p*-value adjustments using the False Discovery Rate for *p* < 0.05 to avoid inflating Type I error due to multiple testing.

^†^Compounds identified with commercial pure standards.
